# Overall survival and short-term efficacy analysis of cervical squamous cell carcinoma with skeletal muscle and ^18^F-FDG PET/CT parameters

**DOI:** 10.1038/s41598-024-55268-2

**Published:** 2024-02-27

**Authors:** Junyu Zhang, Siyu Niu, Xiurong Lu, Ruiying Hu, Zhifang Wu, Suyun Yang, Haiyan Liu

**Affiliations:** 1 Department of Nuclear Medicine, First Hospital of Shanxi Medical University, Taiyuan, China; 2 Collaborative Innovation Center for Molecular Imaging of Precision Medicine, Shanxi Medical University, Taiyuan, China

**Keywords:** Skeletal muscle index, Sarcopenia, ^18^F-FDG PET/CT, Cervical squamous cell carcinoma, Tumor biological metabolism, Cancer imaging, Cervical cancer

## Abstract

2-[^18^F]fluoro-2-deoxy-d-glucose positron emission tomography/computed tomography (^18^F-FDG PET/CT) can provide tumor biological metabolism and skeletal muscle composition information. The aim of this study was to evaluate overall survival (OS) and short-term efficacy of cervical squamous cell carcinoma combining tumor biological metabolism and skeletal muscle composition parameters. Eighty two patients with cervical squamous cell carcinoma were included in the study, who received ^18^F-FDG PET/CT scans before treatment. Clinical characteristics, tumor biological metabolism parameters [standardized uptake value, metabolic tumor volume (MTV), total lesion glycolysis, heterogeneity of tumors, etc.] and body composition parameters were recorded. The survival analysis of cervical squamous cell carcinoma patients was performed by univariate and multivariate analysis. A combined model included clinical indicators, tumor metabolism parameters and sarcopenia was constructed to evaluate OS of patients. According to the Response Evaluation Criteria in Solid Tumours version 1.1, the relationship between sarcopenia with tumor metabolism parameters and short-term efficacy was investigated in subgroup. The results indicate that sarcopenia and high value of the sum of MTV of lesions and metastases (MTV_total_) were poor prognostic factors in patients with cervical squamous cell carcinoma. The combination of sarcopenia, MTV_total_ and clinical factors provided an improved prediction of OS especially in the long term after treatment. Nutritional status of the patients and tumor metabolism may not affect the short-term efficacy of chemoradiotherapy in cervical squamous cell carcinoma patients.

## Introduction

Cervical cancer is one of the most common malignant tumors among women, and consistently a major leading cause of cancer death in women worldwide^1^. Surgery and concurrent chemoradiotherapy (CCRT) based on platinum, are the standard treatments for cervical cancer, but the 5-years survival rate of patients is only 50–65% due to the relatively high recurrence rate after treatment^2,3^. Prognosis and survival of the patients with tumors are associated with nutritional status and body composition according to recent researches^4^. Sarcopenia is defined as a progressive and generalized skeletal muscle disorder, which is a marker of poor nutrition^5^. Sarcopenia has been reported in various cancer including oesophageal cancer, pancreatic cancer, colorectal cancer, and associated with poor prognosis of cervical cancer^6,7^. Computed tomography for measuring skeletal muscle area is an established method and provides specific numerical criteria for assessing sarcopenia^8^. Positron emission tomography/computed tomography (PET/CT) plays a vital role in tumor staging and metabolic activity of many malignant tumors including cervical cancer, which also allows for assessment of the skeletal muscle area and diagnosing sarcopenia at the same time^9,10^. In recent years, many studies have shown that a series of 2-[^18^F]fluoro-2-deoxy-d-glucose (^18^F-FDG) PET/CT semiquantitative indicators including standardized uptake value (SUV), metabolic tumor volume (MTV) and total lesion glycolysis (TLG) have been identified as independent prognostic factors for tumor recurrence and overall survival in cervical cancer^11,12^.

However, few studies have evaluated the correlation between the parameters indicating tumor metabolic activity and prognostic of cervical cancer patients with sarcopenia, even though many studies have demonstrated sarcopenia and PET semiquantitative indicators to be independent poor prognostic factors for survival and curative effect^11,13,14^. Therefore, the study aims to evaluate the effect of sarcopenia combined with PET semiquantitative parameters on the survival and CCRT efficacy of cervical cancer patients.

## Materials and methods

Cervical cancer specimens and anonymized clinical data were obtained from the First Hospital of Shanxi Medical University. Each cervical cancer specimen was reviewed by a pathologist. This study was approved by the Ethical Committee of the First Hospital of Shanxi Medical University and the need for written informed consent was waived.

### Patients

We retrospectively reviewed patients with consecutive cervical cancer who underwent surgery, concurrent cisplatin-based chemoradiotherapy and brachytherapy between March 2018 and March 2023.

**Figure 1:**
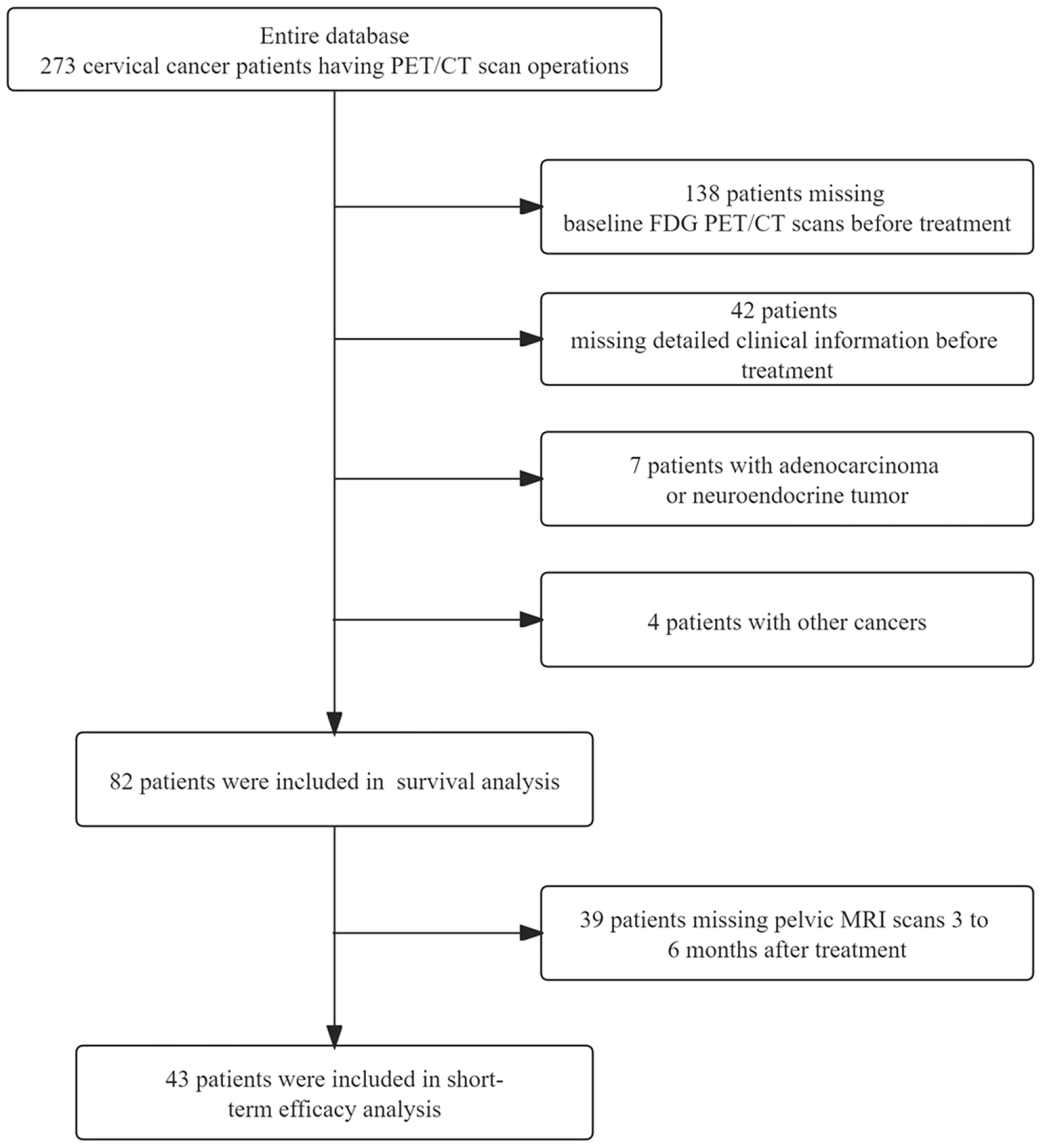
The selection of patient data for analysis.

The following inclusion criteria were applied to determine eligibility: (1) patients with histologic findings of squamous cell carcinoma; (2) patients with the International Federation of Gynecology and Obstetrics 2018 (FIGO 2018) stage IB-IVB; (3) patients with baseline ^18^F-FDG PET/CT scans before treatment; (4) patients with detailed clinical information [liver function indicators, renal function indicators, serum hemoglobin (Hb), serum tumor markers, BMI (body mass index), etc.] before treatment. We excluded patients with alternative histopathologic findings or other cancers and without a baseline examination (Fig. 1). A total of 82 patients met the study criteria in which about 80% of patients received treatment in our hospital, and about 20% did not received treatment in our hospital. The clinical and pathological data are presented in Table 1.

**Table 1:** Baseline clinicopathologic characteristics.

Characteristics	Overall(n = 82)
Clinical indicators	
Age (years), mean ± SD	56.9 ± 12.0
BMI (kg/m^2^), mean ± SD	23.4 ± 3.2
Hb (g/l), mean ± SD	120.5 ± 22.5
Urea (mmol/l), mean ± SD	4.7 ± 1.1
Creatinine (μmol/l), mean ± SD	59.6 ± 11.6
SCC-Ag (ng/ml), median (IQR)	7.2 (12.0)
ALT (U/L), median (IQR)	13 (6.8)
AST (U/L), median (IQR)	18.5 (8.0)
Body composition parameters	
T4MD (HU), mean ± SD	40.5 ± 4.3
T4MI (cm^2^/m^2^), mean ± SD	56.5 ± 8.1
T12MD (HU), mean ± SD	33.3 ± 6.1
T12MI (cm^2^/m^2^), mean ± SD	24.7 ± 3.2
L3MD (HU), mean ± SD	31.6 ± 6.4
L3MI (cm^2^/m^2^), mean ± SD	37.0 ± 5.3
FIGO stage, n (%)	
IB–II	34 (41.5)
III–IVB	48 (58.5)
Lymphatic metastasis, n (%)	52 (63.4)
1 regional lymph node	37 (45.1)
≥ 2 regional lymph node	15 (18.3)
PET/CT metabolic parameters	
SUV_max_, median (IQR)	14.2 (7.9)
MTV, median (IQR)	27.4 (37.5)
TLG, median (IQR)	227.3 (337.4)
MTV_total_, median (IQR)	28.0 (45.0)
TLG_total_, median (IQR)	246.5 (342.9)
Heterogeneity, median (IQR)	0.25 (0.10)
Treatment, n (%)	
Chemoradiotherapy	50 (61.0)
Surgery	6 (7.2)
Surgery + Chemoradiotherapy	8 (9.8)
Unknown	18 (22.0)

*BMI* body mass index, *Hb* serum hemoglobin, *SCC-Ag* sguamous cell carcinoma associated antigen, *ALT* alanine amino transferase, *AST* aspartate amino transferase, *T4MD* skeletal muscle density of level of the fourth thoracic vertebra, *T4MI* skeletal muscle index of level of the fourth thoracic vertebra, *T12MD* skeletal muscle density of level of the twelfth thoracic vertebra, *T12MI* skeletal muscle index of level of the twelfth thoracic vertebra, *L3MD* skeletal muscle density of level of the third lumbar vertebra, *L3MI* skeletal muscle index of level of the third lumbar vertebra, *SUV*_*max*_ maximum value of standardized uptake value, *MTV* metabolic tumor volume, *TLG* total lesion glycolysis, *MTV*_*total*_ the sum of MTV of lesions and metastases, *TLG*_*total*_ the sum of TLG of lesions and metastases, *SD* standard deviation, *IQR* interquartile range.

### Image acquisition and processing

^18^F-FDG PET/CT scanning was performed using the Discovery MI PET/ CT apparatus (GE Healthcare, USA). The radioactive tracer ^18^F-FDG was automatically synthesized by the cyclotron (Sumitomo, Japan) and the ^18^F-FDG chemical synthesis module, and the radioactive tracer purity was guaranteed to be > 99%. The patients fasted for 4 to 6 h before the examination, and their blood sugar levels were within the normal range. ^18^F-FDG was injected intravenously at 3.7 MBq/kg body weight, and then patients rested for 50–70 min after injection. Scans were obtained from the skull base to the midthigh with the following parameters: tube voltage 120 kV, tube current 60–150 mA (automatic adjustment in x–y direction/z direction), noise index 18, pitch 0.984:1, slice thickness 2.75 mm, rotation time 0.5 s; PET scan in list mode, 3 min/bed, 5–7 beds. ^18^F-FDG PET/CT images were reconstructed with iterative reconstruction and displayed on a PET/CT workstation (PET VCAR; GE Healthcare). The PET VCAR software of the post-processing workstation was used to determine the threshold of drawing the edge of tumor by iterative adaptive algorithm to extract the TLG and SUV of the focus (Supplementary Fig. S1). MTV was defined as the TLG dividing by the SUV_mean_^15^. MTV_total_ was defined as the sum of MTV of lesions and metastases. TLG_total_ was also defined in this way. SUV_max_ was defined by the highest pixel value in the region of interest and SUV_mean_ by the average pixel value. Heterogeneity of tumor was expressed by SD/SUV_mean_^12,16^.

The 3.0 T magnetic esonance imaging (MRI) scanner (Skyra: Siemens, Erlangen, Germany) was used of which the scanning range covered the pelvis, and the scanning sequence included conventional fast spin echo (FSE) T2-weighted imaging (T_2_WI), gradient recalled echo (GRE) diffusion-weighted imaging (DWI), and volumetric interpolated body examination dynamic contrast enhancement (VIBE DCE). T_2_WI image scanning parameters were: repetition time (TR) = 4143 ms; echo time (TE) = 85.3 ms; slice thickness = 4 mm; slice spacing = 1 mm; matrix = 384 × 384. The contrast agent (gadoteric acid meglumine, Heng Rui Company, China) was given at 0.2 mmol/kg and an injection flow rate of 2.5 mL/s to obtain the arterial phase (A), intravenous phase (V), and delayed phase (D) images.

### CT body composition analysis

**Figure 2:**
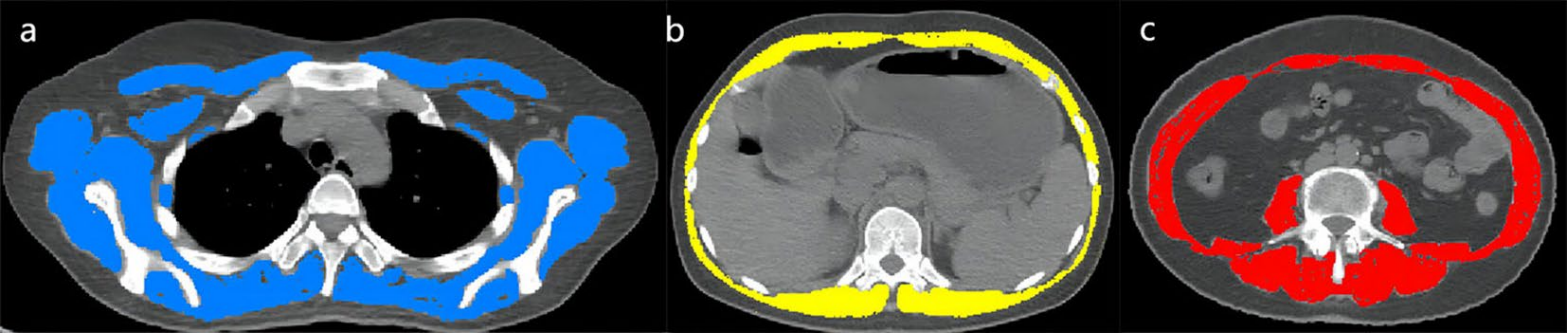
Representative example of skeletal muscle measured (threshold − 29 to 150 HU) on a cross-sectional image at the level of T4, T12, L3 and outline in blue, yellow, red respectively. T4, the fourth thoracic vertebra; T12, the twelfth thoracic vertebra; L3, the third lumbar vertebra.

Sarcopenia measurements were calculated from the CT component of PET/CT. The skeletal muscle area in square centimeter was calculated at the thoracic vertebra T4 level, T12 level and lumbar L3 level by Slice-O-Matic software (version 5.0; TomoVision) (Fig. 2). Hounsfield units (HU) were used to identify skeletal muscle (threshold − 29 to 150 HU)^17^. Skeletal muscle index (SMI) was calculated by the muscle area (cm^2^)/square of height (m^2^). The average HU value of skeletal muscle at each vertebral level was recorded as skeletal muscle density (SMD) of each vertebral level^18^. The third lumbar vertebra (L3) level skeletal muscle index was analyzed to determine the sarcopenia. L3 muscle index (L3MI) cuttoff for sarcopenia was 38.5 cm^2^/m^2^ in females based on previously established consensus^6,19,20^.

### Chemoradiotherapy planning

Patients included in chemoradiotherapy program received intensity-modulated radiotherapy consisting of 6 to 9 coplanar fields using 6 or 10 MV photons. The whole pelvis was irradiated with a dose of 45.0–50.4 Gy (1.8 Gy per fraction). Extended-field radiotherapy was considered for patients with positive pelvic lymph nodes or FIGO stage III–IVA disease. The dose to the involved pelvic lymph nodes was boosted to 59–60 Gy. The prescribed dose for each brachytherapy was 5.0 Gy to point A for 6 sessions. Concurrent chemotherapy consisted of 5 or 6 weekly cycles of intravenous cisplatin (40 mg/m^2^).

### Follow-up information and efficacy evaluation

The follow-up information came from outpatient follow-up review or telephone follow-up. The end of follow-up was the time of last follow-up (July 2023) or death and median follow-up time was 20 months. The short-term curative effect was defined as the lesion condition 3–6 months after treatment by comparison of imaging data before and after treatment (Fig. 5). Short-term efficacy was classified by Response Evaluation Criteria in Solid Tumors version 1.1 (RECIST 1.1) including complete remission (CR), partial remission (PR), stable disease (SD) and progression disease (PD)^21^.

### Statistical analysis

All statistical analyses were performed using the SPSS26.0, GraphPad Prism 9 and R v4.2.3. All statistical tests of hypothesis were two-sided and performed at the 0.05 level of significance.

Categorical variables are expressed as frequencies and percentages. Continuous variables are presented as the mean ± standard deviation (M ± SD) or median (interquartile range, IQR). Student t-test or Mann Whitney U-test was used to compare the differences in continuous variables after the Shapiro–Wilk testing for normality. Categoric variables were compared using the χ^2^ test. For survival analysis, the optimal cutpoints for multiple continuous variables were determined using the maximally selected rank statistics from the “maxstat” R package. The proportional hazard assumption was checked using the Schoenfeld residual test. Survival curves were built using the Kaplan–Meier method and survival differences were assessed using the log-rank test. Then the factors with P value less than 0.05 were included in the multivariate Cox regression with forward stepwise (likelihood ratio), and hazard ratios of factors with statistical differences were calculated. The nomogram was shown for the prediction of 12-, 24- and 36-months overall survival (OS). The performance of the nomogram was evaluated using the concordance index and calibration curve with bootstrap self-sampling method (Supplementary Figs. S2 and S3). Model performance was quantified and visualized using area under the time-dependent receiver operating characteristic (ROC) curve (AUC). The nomogram and AUC curves were drawn using the *rms* R package and *timeROC* R package, respectively.

The association of sarcopenia with short-term curative effect was investigated by logistic regression analysis with enter procedure after adjustment for probable causes of both exposure and outcome. The covariates considered were: FIGO stage and MTV_total_.

### Ethics approval and consent to participate

All procedures performed in studies involving human participants were in accordance with the ethical standards of the institutional research committee and with the 1964 Helsinki Declaration and its later amendments or comparable ethical standards.

## Results

### Baseline characteristics of patients

In this study, overall, 50 patients (61.0%) received chemoradiotherapy and brachytherapy; 6 patients (7.2%) received surgery alone; 8 patients (9.8%) received surgery and chemoradiotherapy with brachytherapy, and 18 (22.0%) patients did not receive any treatment in our hospital.

### Analysis of patient survival outcomes

**Figure 3:**
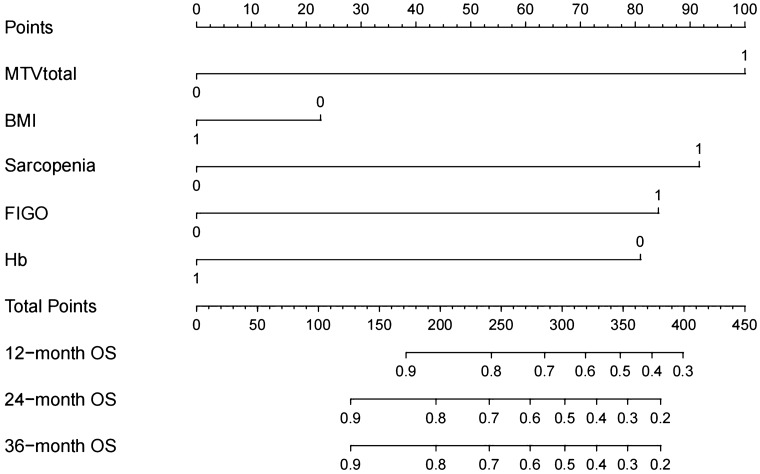
Nomogram: OS for 12 months, 24 months and 36 months. For MTV_total_, FIGO, BMI and Hb, 0 = low and 1 = high. For sarcopenia, 0 = no and 1 = yes. OS, overall survival; MTV_total_, the sum of metabolic tumor volume (MTV) of lesions and metastases; FIGO, the International Federation of Gynecology and Obstetrics 2018 stage; BMI, body mass index; Hb, serum hemoglobin.

**Figure 4:**
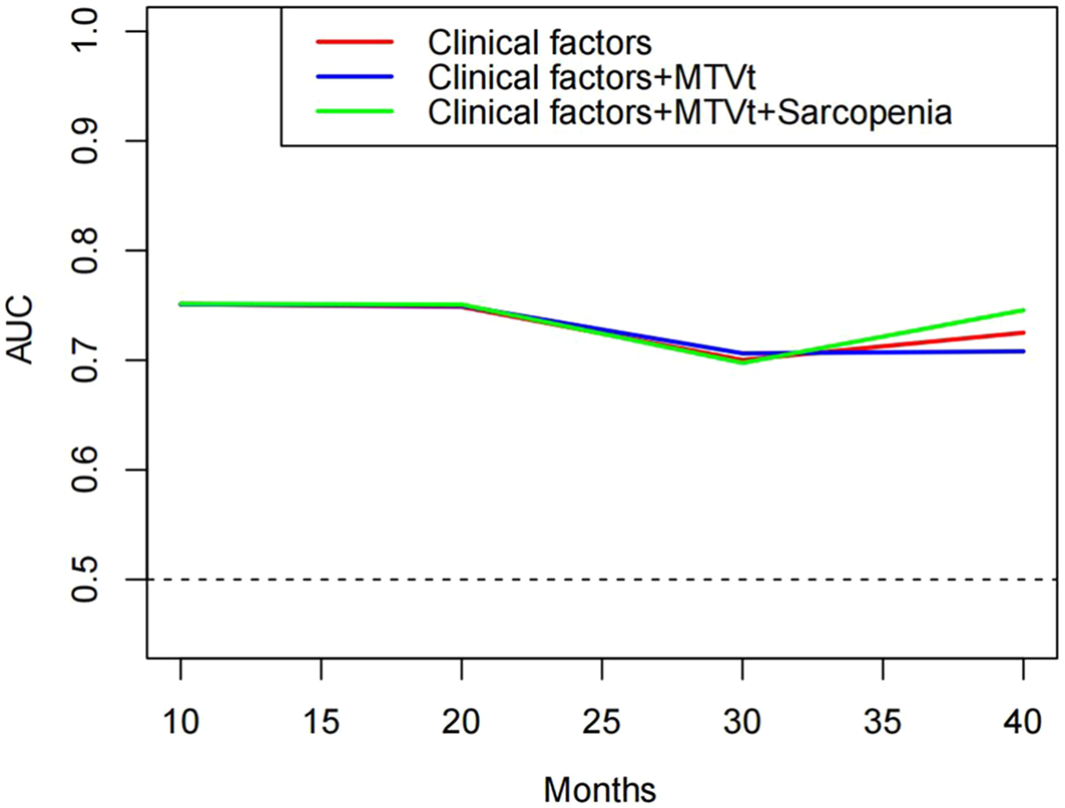
Time-dependent AUC for OS. Clinical factors—FIGO 2018 stage, Hb and BMI; AUC, area under the time-dependent receiver operating characteristic curve; MTVt, the sum of metabolic tumor volume of lesions and metastases (MTV_total_); BMI, body mass index; Hb, serum hemoglobin; FIGO 2018 stage, the International Federation of Gynecology and Obstetrics 2018 stage.

A total of 82 patients were followed for survival outcomes (67 patients survived and 15 patients died). The median follow-up period was 20 (IQR, 8 to 27) months. We selected 1–2 representative parameters from each category of functional indicators and included them in Kaplan–Meier analysis respectively. In Kaplan–Meier analysis, Hb (HR 0.20; 95% CI 0.07–0.55; *P* = 0.005), MTV_total_ (HR 5.03; 95% CI 1.51–16.75; *P* < 0.001), sarcopenia (HR 0.34; 95% CI 0.12–0.94; *P* = 0.0495) and FIGO stage (HR 12.25; 95% CI 4.44–33.78; *P* = 0.002) were significantly associated with OS. In the multivariate analysis, significance was maintained for MTV_total_ (HR 5.54; 95% CI 1.94–15.80; *P* = 0.001) and sarcopenia (HR 0.30; 95% CI 0.09–0.96; *P* = 0.042). Other covariates included in the stepwise regression models were not significant (value of HR not shown) (Table 2 and Supplementary Fig. S4). We scored MTV_total_, sarcopenia, and other clinical indicators, and created a nomogram based on the impact of various functional indicators on patient OS (Fig. 3). The nomogram showed MTV_total_ and sarcopenia have higher scores in predicting OS. In 10–40 months, the concordance index were greater than 0.7 and the calibration curve showed good agreement between predicted probabilities and actual observations (Supplementary Figs. S2, S3). A clinical model was built using clinical factors (FIGO stage, Hb and BMI) to predict the OS. Unexpectedly, the model containing the MTV_total_ perform similarly compared to the clinical model alone for OS with AUC of 0.751, 0.750, 0.706, 0.708 and AUC of 0.751, 0.749, 0.700, 0.725 at 10, 20, 30, 40 months follow-up, respectively. In addition, the fully combined model (clinical factors, MTV_total_, and sarcopenic status) was able to better predicate the OS especially in the 40 months of follow-up with AUC of 0.752, 0.751, 0.698, 0.746 (Fig. 4).

**Table 2:** Univariable and multivariable analyses of clinical, PET/CT semiquantitative and body composition parameters for OS.

Variable	Univariate analysis		Multivariate analysis	
	HR (95% CI)	*P* value	HR (95% CI)	*P* value
Age				
< 68 (years)	Reference			
≥ 68 (years)	2.22 (0.60–8.18)	0.132		
Hb				
< 127 (g/l)	Reference			
≥ 127 (g/l)	0.20 (0.07–0.55)	0.005	–	0.100
Urea				
< 3.35 (mmol/l)	Reference			
≥ 3.35 (mmol/l)	0.34 (0.05–2.23)	0.074		
ALT				
< 13 (U/L)	Reference			
≥ 13 (U/L)	0.49 (0.18–1.37)	0.168		
SUV_max_				
< 8.81	Reference			
≥ 8.81	3.24 (0.72–14.57)	0.125		
MTV_total_				
< 57.40	Reference		Reference	
≥ 57.40	5.03 (1.51–16.75)	< 0.001	5.54 (1.94–15.80)	0.001
SCC-Ag				
< 14.26 (ng/mL)	Reference			
≥ 14.26 (ng/mL)	1.99 (0.625–6.40)	0.179		
BMI				
< 19.92 (kg/m^2^)	Reference			
≥ 19.92 (kg/m^2^)	0.28 (0.05–1.60)	0.017	–	0.514
Sarcopenia				
Yes	Reference		Reference	
No	0.34 (0.12–0.94)	0.0495	0.30 (0.09–0.96)	0.042
FIGO stage				
IB–II	Reference			
III–IVB	12.25 (4.44–33.78)	0.002	–	0.143
Lymphatic metastasis				
Yes	Reference			
No	0.36 (0.13 to 1.02)	0.097		

*OS* overall survival, *Hb* serum hemoglobin, *ALT* alanine amino transferase, *SUV*_*max*_ maximum value of standardized uptake value, *MTV*_*total*_ the sum of metabolic tumor volume of lesions and metastases, *SCC-Ag* sguamous cell carcinoma associated antigen, *BMI* body mass index, *FIGO stage* the international federation of gynecology and obstetrics 2018 stage.

### Analysis of short-term efficacy

**Figure 5:**
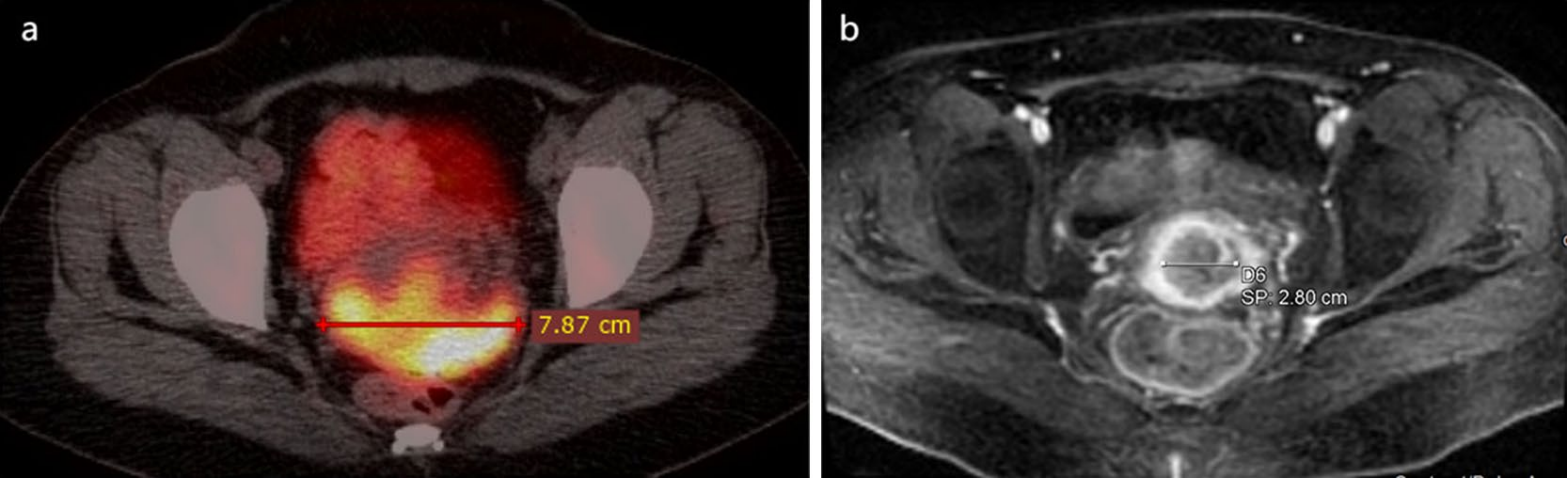
Images of a 31-year-old patient with (FIGO 2018) stage IIIC squamous cell carcinoma before and after treatment. (**a**) Axial fusion PET/CT images of pelvic before treatment. (**b**) Axial T2WI MRI image of pelvic 3 months after treatment.

43 of all cervical cancer patients underwent enhanced pelvic MRI scans 3 to 6 months after treatment. No statistical differences were observed between the subgroup and entire study population in routine clinical indicators, PET/CT tumor metabolism parameters, and body composition parameters (Table S1). Therefore, the efficacy analysis still used the cutoff values of each variable of survival analysis. According to RECIST 1.1, 13 patients (30.2%) had achieved CR, 26 (60.5%) had achieved PR, 3 (7.0%) had SD, and 1(2.3%) had PD 3–6 months after treatment. We defined the objective response as CR + PR (n = 39, 90.7%) and the non-response as SD + PD (n = 4, 9.3%) (Table 3 and Fig. 5). The univariate analysis showed no significance was observed for MTV_total_ (OR 2.90; 95% CI 0.36–23.39), sarcopenia (OR 0.86; 95% CI 0.11–6.72) and FIGO stage (OR 2.85; 95% CI 0.27–29.84) of cervical cancer patients (*P* > 0.05). When adjustments were made for MTV_total_ and FIGO stage, the multivariate analysis showed that no statistical significance was also observed for sarcopenia (OR 0.82; 95% CI 0.09–7.79) (*P* > 0.05) (Table 3).

**Table 3:** Univariate and multivariate analysis of short-term efficacy.

Variable	Objective response	Non-response	Univariate analysis		Multivariate analysis	
	N = 39 (%)	N = 4 (%)	OR (95% CI)	*P* value	OR (95% CI)	*P* value
MTV_total_						
< 57.40	29 (74.4)	2 (50.0)	Reference		Reference	
≥ 57.40	10 (25.6)	2 (50.0)	2.90 (0.36–23.39)	0.317	2.26 (0.18–28.11)	0.527
Sarcopenia						
Yes	18 (46.2)	2 (50.0)	Reference		Reference	
No	21 (53.8)	2 (50.0)	0.86 (0.11–6.72)	0.883	0.82 (0.09–7.79)	0.860
FIGO stage						
IB–II	19 (48.7)	1 (25.0)	Reference		Reference	
III–IVB	20 (51.3)	3 (75.0)	2.85 (0.27–29.84)	0.382	1.86 (0.11–30.25)	0.663

*MTV*_*total*_ the sum of metabolic tumor volume of lesions and metastases, *FIGO stage* the international federation of gynecology and obstetrics 2018 stage.

## Discussion

Most patients in this study received surgery and /or concurrent platinum-based chemoradiotherapy followed by brachytherapy. We investigated the prognostic value of clinical indicators, ^18^F-FDG PET/CT tumor metabolic parameters and body composition parameters in patients with cervical squamous cell carcinoma and found that sarcopenia (low L3MI value) and high MTV_total_ were prognostic markers for poor OS. Furthermore, improved prognostication of OS was observed when sarcopenia status was combined as opposed to clinical variables only or clinical variables combined with MTV_total_. However, these parameters were not associated with short-term efficacy in outcome prediction.

Some prior studies have separately discussed the predictive value of sarcopenia and tumor metabolism parameters for OS in various types of tumors, but very few studies have evaluated the predictive prognostic value of body composition parameters combined with tumor metabolic parameters both from PET/CT, especially in cervical cancer. These studies found that sarcopenia was negatively associated with long-term outcomes in rectal, gastric, esophageal cancer and other solid tumors^6,22,23^. In addition, two previous studies have shown that sarcopenia predicts poor prognosis for patients with stage II–III cervical cancer on concurrent chemoradiotherapy showing significantly lower OS (HR 2.473, *P* = 0.020; HR 6.035, *P* = 0.001), which is consistent with our findings^13,24^. Although SUV is widely used to evaluate the biological activity of malignancies, SUV_max_ may vary depending on the reconstruction algorithm or patient preparation before scan^25^. Therefore, it is important to evaluate tumor biological activity with multiple indicators (MTV, TLG and others). Many studies showed that SUV_max_, MTV, TLG and other parameters of the biological activity of tumor in PET images were closely related to the OS of patients with cervical cancer^26–28^. However, in this study, significance was observed for MTV_total_ but not for SUV_max_. In light of these findings, current research speculates that the reduced survival rate we observed in patients with sarcopenia could result from decreased energy reserves, disruption of body energy balance due to malnutrition^29,30^, and decreased survival observed in patients with high MTV may be due to metabolic activity of tumor cells and high invasiveness of tumors^31^. Therefore, the amount of muscle and metabolic activity of tumors can be quantified using the PET/CT directly so that we can combine these two indicators to evaluate the prognosis of patients with cervical cancer more effectively. This inference has been confirmed in some tumors. In two studies on esophagogastric and early‑stage adenocarcinoma esophageal cancer, clinical parameters integrated with PET/CT metabolic parameters and sarcopenia improve outcome prediction in a complementary fashion^10,32^. However, there are also some studies that contradict to our founding. Some researchers found the MTV, TLG and sarcopenia were not independent prognostic factors for OS in elderly mantle cell lymphoma^33^. Additionally, we included clinical factors as covariates in the multivariate analysis for OS, and no significance was observed in the clinical factors. Similar results have been reported in a study on cervical cancer, in which association of pretreatment SUV_max_ of focus and SCC-Ag with FIGO 2018 stage and relationship to prognosis were studied. The founding showed significance was observed for FIGO 2018 stage but not for SCC-Ag and other clinical factors^34^.

Two research results showed that the percentages of patients with cervical cancer who received concurrent chemoradiotherapy achieved objective responses after two months were 98% (squamous cell carcinoma) and complete remission were 86.2% respectively, which were similar to our results^35,36^. Residual tumor is common after treatment especially for those with large masses, which imperils patient survival and control of tumor progression. Researchers have discovered that the smaller the residual tumor, the better the prognosis for patient survival^37^. In our study about short-term efficacy, no statistical significances were observed for sarcopenia and MTV_total_. The founding suggested that patients with sarcopenia or high MTV_total_ have non-inferior short-term efficacy compared to patients with good nutritional status or low MTV_total_ in cervical squamous cell carcinoma.

Nevertheless, this study has some limitations that must be acknowledged. Firstly, given the small sample size of squamous cell carcinoma cases and only a single pathological type included, the generalizability of the results may be limited. Secondly, there are few studies on evaluating the prognosis of cervical cancer by combining tumor metabolism and body composition parameters from PET/CT, so similar studies on other tumors were cited to support our results. Thirdly, tumors are sometimes difficult to separate from urine in the bladder, because they have similar signal intensities on PET images. To solve this problem, the ROI of tumors were manually corrected layer by layer on the axial position of the PET/CT fusion image resulting in a certain level of subjectivity in the analysis process. Finally, our patients only had baseline PET/CT data so that we cannot evaluate the impact of changes of skeletal muscle index and tumor metabolic parameters after treatment on prognosis of patients and a few of patients receive no therapy in our hospital, whose treatments were unknown.

## Conclusion

This study demonstrates that sarcopenia and high MTV_total_ are poor prognostic factors in a cohort of patients with cervical squamous cell carcinoma. The combination of sarcopenia, MTV_total_ and clinical factors provides improved prediction of OS, especially in the long term after treatment, compared to model with only clinical factors and clinical factors combining with MTV_total_. Patients with cervical squamous cell carcinoma, who have sarcopenia and a high MTV_total_, demonstrate a non-inferior clinical response to timely chemoradiotherapy, suggesting that the nutritional status of the patients and tumor metabolism may not affect the short-term efficacy of chemoradiotherapy.

### Supplementary Information

Supplementary Information.

## Data Availability

Data generated or analyzed during this study are included in this published article. The raw data for this study were generated at First Hospital of Shanxi Medical University. The data supporting the findings of this study are available from the corresponding author upon request.
