# Supplementary material for: Overall survival and short-term efficacy analysis of cervical squamous cell carcinoma with skeletal muscle and 18F-FDG PET/CT parameters
Source: Sci Rep. 2024 Feb 27;14:4809. doi: 10.1038/s41598-024-55268-2 (PMC10899580; doi:10.1038/s41598-024-55268-2)
Supplement: Supplementary file 1 — Supplementary Information. [file 41598_2024_55268_MOESM1_ESM.doc]

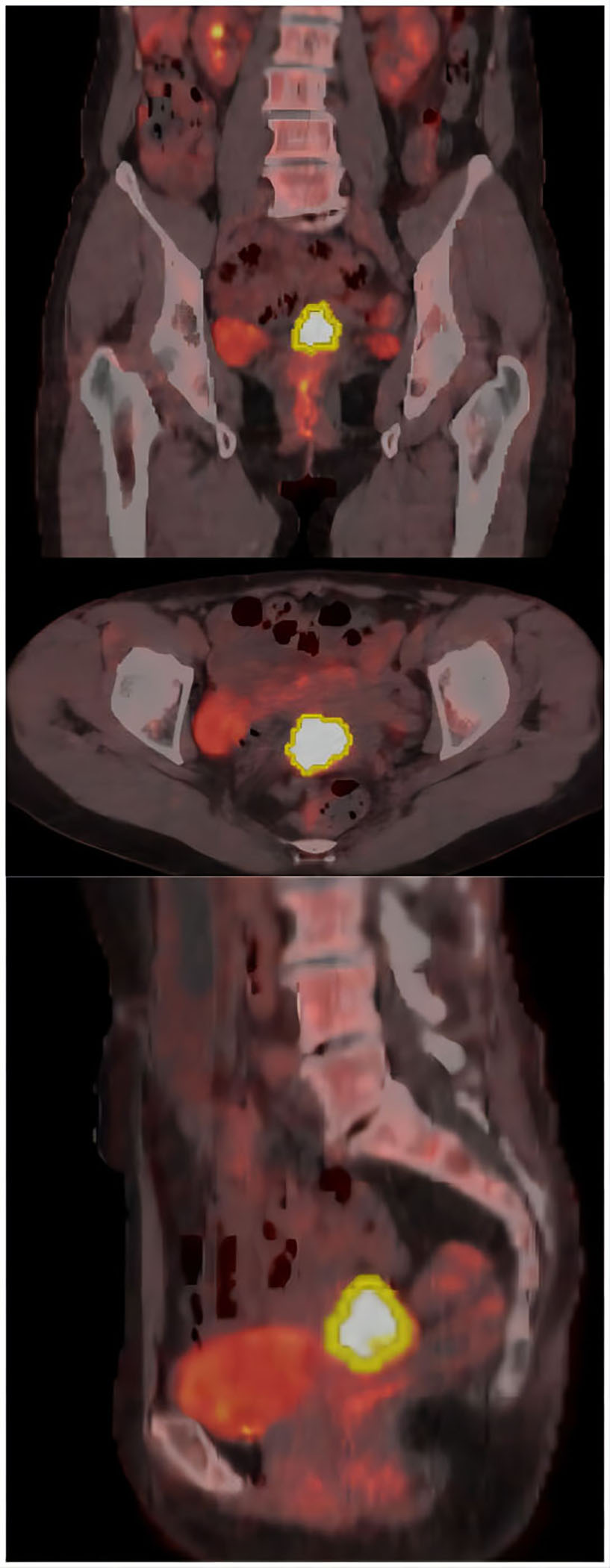


Supplementary Fig S1. Fused PET/CT images on axial, sagittal and coronal planes, respectively, showing focus of cancer with ROI by iterative adaptive algorithm of PET VCAR software.


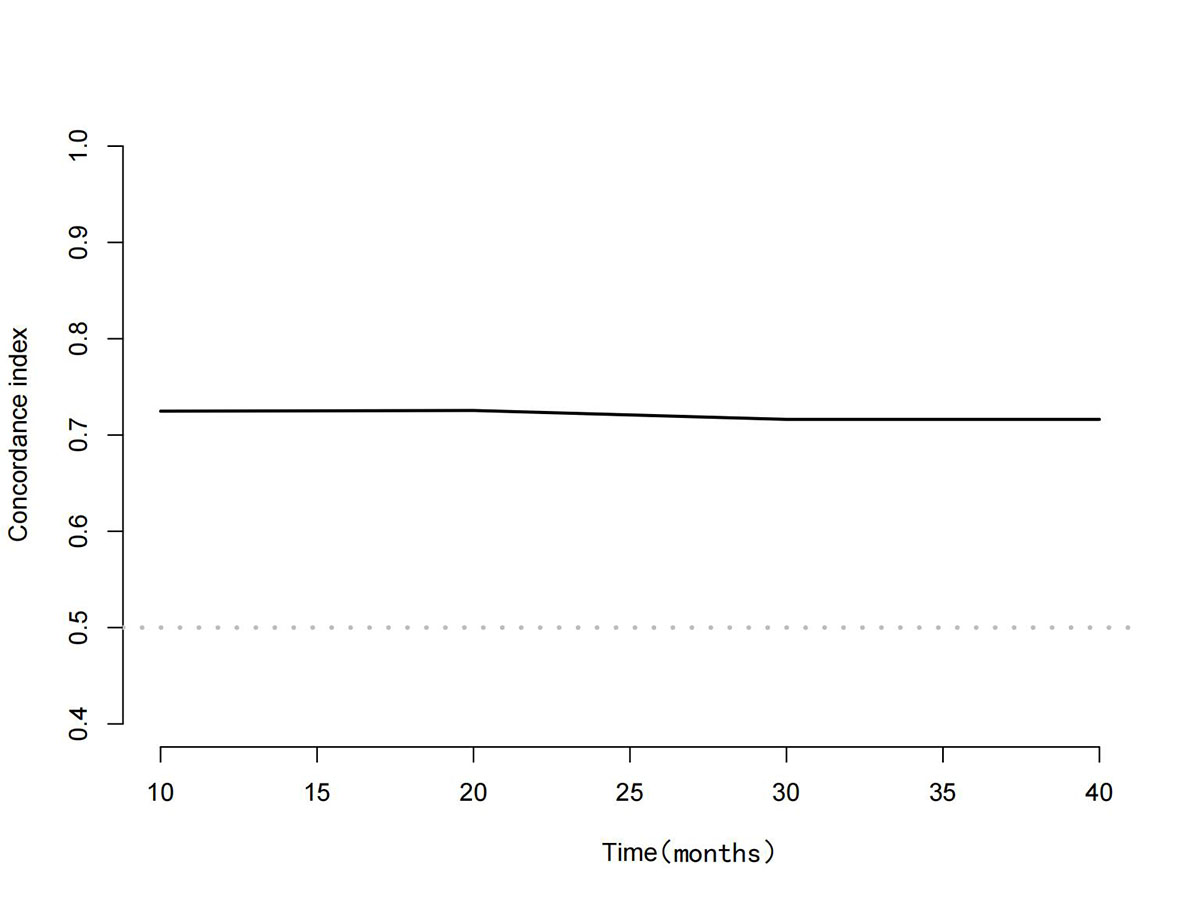


Supplementary Fig S2. Concordance index for 10-40 months.


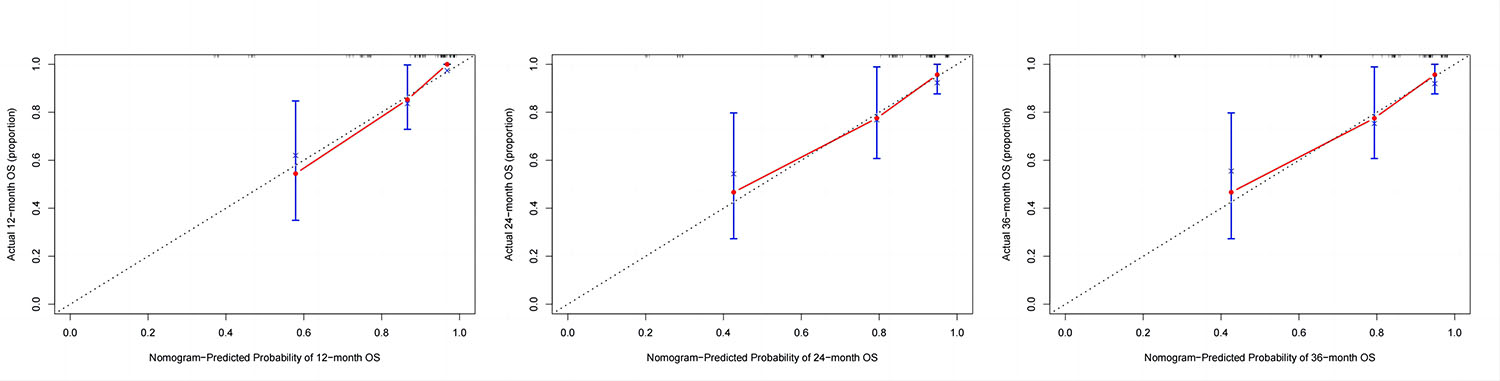


Supplementary Fig S3. Calibration curve for 12-month, 24-month and 36-month OS. The Y-axis represents actual survival, and the X-axis represents nomogram-predicted survival.


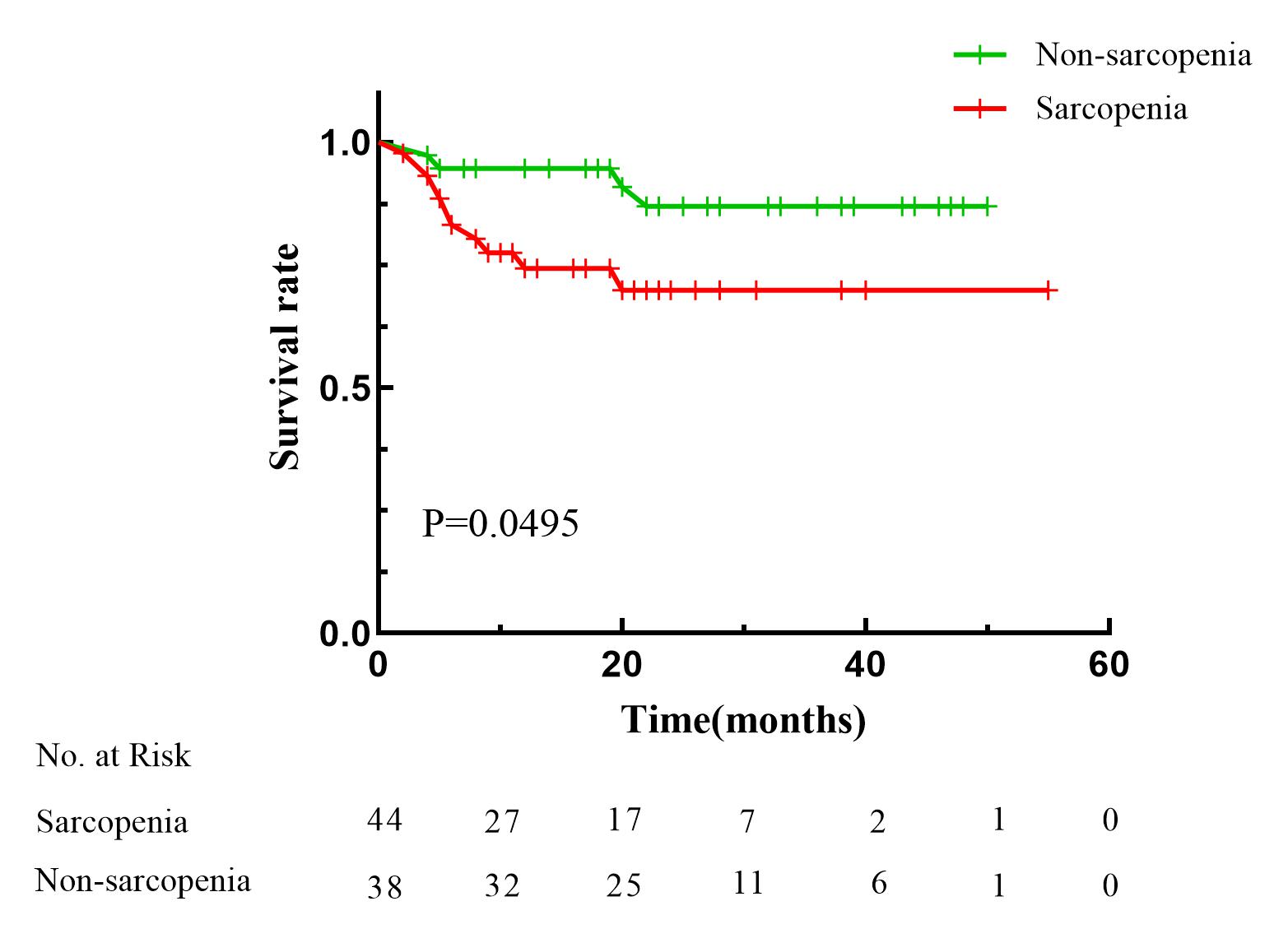


Supplementary Fig S4. Kaplan-Meier curves show overall survival rate in patients according to sarcopenia status.

| Characteristics | Overall (n = 82) | Subgroup (n= 43) | P-value |
| --- | --- | --- | --- |
| Age (years), mean±SD | 56.9±12.0 | 56.2±13.1 | 0.740 |
| BMI (kg/m^2^), mean±SD | 23.4±3.2 | 24.0±2.9 | 0.306 |
| Hb (g/l), mean±SD | 120.5±22.5 | 118.1±25.8 | 0.595 |
| Urea (mmol/l), mean±SD | 4.7±1.1 | 4.8±1.2 | 0.677 |
| Creatinine (μmol/l), mean±SD | 59.6±11.6 | 61.4±13.4 | 0.441 |
| SCC-Ag (ng/ml), median (IQR) | 7.2 (12.0) | 9.48 (12.5) | 0.279 |
| ALT (U/L), median (IQR) | 13 (6.8) | 13 (9.5) | 0.791 |
| AST (U/L), median (IQR) | 18.5 (8.0) | 19 (12.5) | 0.721 |
| **Body composition parameters** |  |  |  |
| T12MD (HU), mean±SD | 33.3±6.1 | 33.4±5.8 | 0.909 |
| T12MI (cm^2^/m^2^), mean±SD | 24.7±3.2 | 24.8±3.5 | 0.818 |
| T4MD (HU), mean±SD | 40.5±4.3 | 40.3±4.5 | 0.852 |
| T4MI (cm^2^/m^2^), mean±SD | 56.5±8.1 | 56.3±6.3 | 0.911 |
| L3MD (HU), mean±SD | 31.6±6.4 | 31.8±6.4 | 0.878 |
| L3MI (cm^2^/m^2^), mean±SD | 37.0±5.3 | 37.8±5.5 | 0.447 |
| **FIGO stage, n (%)** |  |  |  |
| IB-II | 34 (41.5) | 20 (46.5) |  |
| III-IVB | 48 (58.5) | 23 (53.5) | 0.501 |
| **Lymphatic metastasis, n (%)** | 52 (63.4) | 26 (60.5) | 0.746 |
| 1 regional lymph node | 37 (45.1) | 18 (41.9) |  |
| ≥2 regional lymph node | 15 (18.3) | 8 (18.6) | 0.861 |
| **PET/CT metabolic parameters** |  |  |  |
| SUVmax, median (IQR) | 14.2 (7.9) | 14.1 (6.3) | 0.813 |
| MTV, median (IQR) | 27.4 (37.5) | 29.0 (38.6) | 0.352 |
| TLG, median (IQR) | 227.3 (337.4) | 238.3 (288.0) | 0.437 |
| MTV_total_, median (IQR) | 28.0 (45.0) | 29.7 (40.7) | 0.418 |
| TLG_total_, median (IQR) | 246.5 (342.9) | 252.9 (292.6) | 0.573 |
| Heterogeneity, median (IQR) | 0.25 (0.10) | 0.26 (0.10) | 0.414 |

Table S1. Characteristics of subgroup and overall subjects
